# *Pseudomonas aeruginosa* inhibits quorum-sensing mechanisms of soft rot pathogen *Lelliottia amnigena* RCE to regulate its virulence factors and biofilm formation

**DOI:** 10.3389/fmicb.2022.977669

**Published:** 2022-08-23

**Authors:** Chintan Kapadia, Rinkal Kachhdia, Susheel Singh, Kelvin Gandhi, Peter Poczai, Saleh Alfarraj, Mohammad Javed Ansari, Abdul Gafur, R. Z. Sayyed

**Affiliations:** ^1^Department of Plant Molecular Biology and Biotechnology, ASPEE College of Horticulture and Forestry, Navsari Agricultural University, Navsari, India; ^2^Food Quality Testing Laboratory, N. M. College of Agriculture, Navsari Agricultural University, Navsari, India; ^3^Finnish Museum of Natural History, University of Helsinki, Helsinki, Finland; ^4^Zoology Department, College of Science, King Saud University, Riyadh, Saudi Arabia; ^5^Department of Botany, Hindu College, Moradabad (Mahatma Jyotiba Phule Rohilkhand University Bareilly), Moradabad, India; ^6^Sinarmas Forestry Corporate Research and Development, Perawang, Indonesia; ^7^Department of Microbiology, PSGVP Mandals, S I Patil Arts, G B Patel Science and STKV Sangh Commerce College, Shahada, India

**Keywords:** cyclic dipeptides, diketopiperazine, *Lelliottia amnigena*, *P. aeruginosa* RKC1, quorum quenching, soft rot

## Abstract

The quorum-sensing (QS) cascade is responsible for the colonization and phenotypic behavior of the pathogenic organism and the regulation of diverse signal molecules. The disruption of the quorum-sensing system is an effective strategy to overcome the possibility of antibiotic resistance development in the pathogen. The quorum quenching does not kill the microbes. Instead, it hinders the expression of pathogenic traits. In the present experiment, *Pseudomonas aeruginosa* RKC1 was used to extract the metabolites responsible for quorum-sensing inhibition in soft rot pathogen *Lelliottia amnigena* RCE. During the initial screening, *P. aeruginosa* RKC1 was found to be most promising and inhibits violacein of *Chromobacterium violaceum* MTCC2656 pyocyanin, swarming-swimming motility of *P. aeruginosa* MTCC2297. The characterization of metabolites produced by the microbes which are responsible for quorum-sensing inhibition through GC-MS is very scarce in scientific literature. The ethyl acetate extract of *P. aeruginosa* RKC1 inhibits biofilm formation of *L. amnigena* RCE while inhibiting growth at higher concentrations. The GC-MS analysis suggested that Cyclic dipeptides (CDPs) such as Cyclo (L-prolyl-L-valine), Cyclo (Pro-Leu), and Cyclo(D-phenylalanyl-L-prolyl) were predominantly found in the ethyl acetate extract of the *P. aeruginosa* RKC1 (93.72%). This diketopiperazine (DKPs) exhibited quorum-sensing inhibition against the pathogen in liquid media during the active growth phase and regulated diverse metabolites of the pathogen. Moreover, the metabolites data from the clear zone around wells showed a higher concentration of DKSs (9.66%) compared to other metabolites. So far, very few reports indicate the role of DKPs or CDPs in inhibiting the quorum-sensing system in plant pathogenic bacteria. This is one such report that exploits metabolites of *P. aeruginosa* RKC1. The present investigation provided evidence to use quorum-sensing inhibitor metabolites, to suppress microbes' pathogenesis and thus develop an innovative strategy to overcome antibiotic resistance.

## Introduction

The uses of various anti-microbial agents lead to the death of diverse microbes. On the other hand, repeated use of such compounds develops resistance. The microorganisms quickly evolved by either changing the target molecules or change in the receptor's proteins. These survivals of fittest principles against stressful environments always ask the scientist to develop novel antibiotic agents or strategies to kill them (Wang et al., 2018). To combat pathogens, nanoparticle alone or in combination with antibiotics agents or CRISPR/Cas9 systems made it possible to control pathogens (Kapadia et al., 2021; Wan et al., 2021). To control pathogens, one can use alternative strategies like anti-biofilm peptides, competitive microbial agents, and antimicrobial peptides. To avoid the development of multi-drug resistant microbes, there is a need to develop novel approaches to control the expression of pathogenesis-related traits of bacteria while not affecting the growth of the pathogen (Laxminarayan et al., 2013; Passari et al., 2017). These virulence factors are governed by the quorum-sensing mediated cascade.

Bacteria are unicellular organisms and hence require communication signals to modify their phenotype in response to environmental conditions. Quorum sensing involves the secretion of autoinducers (N-acyl Homoserine lactone), small peptides, diffusible signal factors (DSFs), and their detection followed by transcriptional regulation. The binding of ligands or metabolites to the receptors brings out changes in the gene expression pattern. Once sufficient cell density is attained, the concentration of autoinducers simultaneously leads to gene expression modulation. Bioluminescence in *Vibrio* sp., Biofilm formation in *Pseudomonas aeruginosa* and *Ralstonia solanacerum* (Flavier et al., 1997), violacein production in *C. violaceum* (McLean et al., 2004), and virulence factor synthesis in *P. aeruginosa* (Nadal Jimenez et al., 2012) are governed by the quorum-sensing system. One can target auto-inducers by enzymatically hydrolyzing or modifying the signal molecules with the help of acylase, lactonase, and oxidoreductase, thus controlling the quorum-sensing system (Dong and Zhang, 2005; Tan et al., 2013; Fetzner, 2015). While in recent years, scientists have tried to isolate QS inhibitors (QSIs) metabolites from diverse microorganisms such as *P. aeruginosa* (Holden et al., 1999; Almohaywi et al., 2018), *Streptomyces griseorubens* (Çetinkaya et al., 2020), S*treptomyces coelicoflavus* (Hassan et al., 2016), *Serratia marcescens* (Padmavathi et al., 2014), *Streptomyces cavourensis* (Kaaniche et al., 2020), and *Exiguobacterium indicum* (Singh et al., 2017) against various pathogens. The compounds isolated from such bacteria competitively bind to the available receptor site in the pathogen and thus suppress the pathogenicity (Sun et al., 2016). There have been no previous reports indicating the use of bacterial extracts to control the pathogenesis of soft rot-causing pathogen *Lelliottia amnigena* specifically in India.

Bacterial soft rot disease is caused by *Dickeya* spp., *Pectobacterium* spp., *L. amnigena*, and *Entrobacter* spp. (Charkowski, 2018) and affects various horticultural crops during storage, transportation, and growing seasons (Hadizadeh et al., 2019). *L. amnigena* is a causative agent of soft rot of potatoes (Abd Elhafeez and Sayed, 2018) and onion bulb decay in China (Liu and Tang, 2016). There is significant emergence of *L. amnigena* infection in the world, which leads to economic losses in horticultural crops (Liu and Tang, 2016; Osei et al., 2021). Surprisingly, the infection processes of these pathogens are also signaling dependent; hence, a quorum-sensing inhibition strategy could be employed to control the virulence of the pathogens. Previous studies indicated the use of ethyl acetate extracts to isolate QSI compounds (Çetinkaya et al., 2020; Kaaniche et al., 2020).

The heterocyclic compound, 1*H*-pyrrole-2-carboxylic acid from *Streptomyces* spp., inhibits biofilm formation and pyocyanin production in *P. aeruginosa* PAO1 (Hassan et al., 2016). Similarly, ethyl acetate extract of *Bacillus pumilus* S8-07 inhibits the biofilm of *P. aeruginosa* PAO1 (Nithya et al., 2010). Moreover, DKPs found in the ethyl acetate extract of *Brevibacterium casei* inhibit biofilm formation as well as inhibit the synthesis of a virulence factor in *P. aeruginosa* (Rashiya et al., 2021), and 3-Benzyl-Hexahydro-Pyrrolo[1,2-a]Pyrazine-1,4-Dione from *E. indicum* inhibits biofilm formation in *P. aeruginosa* PAO1 and *P. aeruginosa* PAH without affecting the growth (Singh et al., 2017). DKPs such as cyclo(Trp-Ser) of *Rheinheimera aquimaris* inhibit QS-mediated virulence traits in *P. aeruginosa* PA01 (Sun et al., 2016), while DKPs such as cyclo(Ala-Val), cyclo(Pro-Tyr), and cyclo(Phe-Pro) from *P. aeruginosa* inhibit virulence phenotypes of *Serratia liquefaciens* (Holden et al., 1999). Cyclo (L-Pro-L-Pro) and cyclo (D-Pro-D-Pro) are cyclodipeptides formed through cyclodehydration of two amino acids. They are produced naturally by various microorganisms which possess diverse biological properties. These dipeptides suppress pathogenesis in tobacco by triggering stomatal closure, induction of hormone synthesis, and secretion of pathogen-related proteins. It was shown that Gram-negative bacteria produce diketopiperazine which has the ability to bind with LuxR receptors and subsequently either activate or suppress the quorum-sensing system (Holden et al., 1999). There are few reports, yet research is necessary to support the hypothesis that small cyclic dipeptides can target receptor binding sites and thus regulate virulence factors. The present study focused on screening quorum-sensing inhibiting microbes using *C. violaceum* MTCC2656 and consequently evaluated its anti-biofilm activity and suppression of pathogenesis-related traits. The bacterial extracts were used to evaluate their potentiality to inhibit the quorum-sensing system of soft rot causing pathogen *L. amnigena* and extracts were further characterized using GC-MS for identification of potential metabolites.

## Materials and methods

### Screening of soil microorganisms for the production of QS inhibitors

#### Microorganisms used in the study

*C. violaceum* MTCC2656 and *P. aeruginosa* MTCC2297 were purchased from Microbial Type Culture Collection (MTCC), Institute of Microbial Technology (Im-Tech), Chandigarh, India. In a sterilized container, the samples were collected from rhizospheric soil from Navsari Agricultural University, Navsari, India. one gram of sample was mixed with 10 ml sterilized ddH_2_O, and then the suspension was serially diluted from 10^−3^ to 10^−7^. Three plates were spread and incubated from each dilution at 28°C for 48 h. The distinct colony was picked up and sub-cultured on NA media (Hi-media) to obtain pure organisms. The pure colonies were stored as glycerol stock (50% V/V) at −20°C until further use. *L. amnigena* RCE (MZ712952) was isolated from the potato tuber and available in the lab from previous experiments (Kachhadia et al., 2022). In brief, the rotten potato tuber was purchased from the market and surface sterilized using 0.1% mercury chloride for 5 min. The soft jelly-like part was placed on NA and incubated at 28°C for 24 h (Kachhadia et al., 2022).

#### Plate incubation assay for screening of microbes

Monitor strain, *C. violaceum* MTCC2656, was used to assess the quorum quenching potential of the isolates. The 24 h old culture of monitor strain was spread evenly on the nutrient agar plate (Hi-media, India), and isolates were spot inoculated on the surface of the agar. The plates were incubated at 28°C overnight. The colorless area around the viable organisms indicated the isolates' quorum-sensing inhibitory (QSI) activity (Ma et al., 2018).

#### Extraction of bacterial crude extract

Isolates that showed positive results during plate incubation assay were exploited for their metabolites through solvent-solvent extraction. The 100 μl of 24 h old bacterial culture was inoculated in 20 ml Luria–Bertani Broth (LB) (Hi-Media, India) and incubated at 28°C; 150 rpm for 24 h. The culture suspension was centrifuged at 8,000 rpm for 15 min, and cell-free supernatant was mixed with an equal volume of ethyl acetate (Sigma-Aldrich, USA). The organic phase was dispensed in a separate tube, and extraction was repeated three times with ethyl acetate. The entire ethyl acetate fractions were mixed and evaporated under a rotary vacuum evaporator. The dry crude extracts were collected in a separate tube containing Dimethyl Sulfoxide (DMSO) and stored at −20 °C for further use (Nithya et al., 2010).

### Evaluation of anti-quorum-sensing potential of crude extracts

#### Agar well diffusion assay

The 24-h-old culture of monitor strain *C. violaceum* MTCC2656 was spread evenly on the LA (Hi-Media, India), and wells were formed using an 8-mm sterile cork borer. The plates were incubated at 28°C for 24 h, and inhibitory activity was monitored by observing the colorless zone around the wells. The 100 μl (1 mg) of crude extracts were poured into the wells, and DMSO containing well was considered a negative control.

#### Violacein inhibition assay

The 24 h grown culture of *C. violaceum* MTCC2656 was supplemented with different concentrations of crude extract (Stock; 10 mg/ml) and incubated at 28°C and 150 rpm for 24 h. The culture was centrifuged to remove supernatant, and the pellet was mixed with DMSO. The DMSO-containing suspension was centrifuged, and the supernatant was analyzed spectrophotometrically (Shimadzu Europe-UV-2600) at 585 nM. The DMSO with untreated monitor strain pellet was considered negative control (Venkatramanan et al., 2020). The experiment was repeated three times to eliminate error, and the inhibition percentage was calculated as follows:


$$\begin{array}{l} \text{Violacein inhibition \%}=\\ \frac{\text{OD}585\text{ofcontrol}-\text{OD}585\text{oftest}}{\text{OD}585\text{ofcontrol}}\times 100 \end{array}$$

Where, OD585 control and OD585 test are the absorbance of DMSO+monitor strain and DMSO+ crude extract + monitor strain, respectively.

#### Pyocyanin inhibition assay using *P. aeruginosa* MTCC2297

The 100 μl (1 mg) crude extracts of the bacteria were supplemented with 10 ml LB media containing monitor strain (OD^600^; 0.400) and incubated at 28°C for 24 h at 150 rpm. The culture was centrifuged to separate cells, and the supernatant was mixed with 6.0 ml chloroform (Sigma-Aldrich). The chloroform layer containing pyocyanin was acidified using 2 ml of 0.2 N HCL (Hi-media). The OD of the pink layer was measured at 520 nM (Shimadzu UV-2600, Japan) using 0.2 N HCL as the blank (Malešević et al., 2019). Pyocyanin inhibition percentage was calculated using the following formula:


$$\begin{array}{l} \text{Pyocyanin inhibition\%}=\\ \frac{\text{OD}520\text{ofcontrol}-\text{OD}520\text{oftest}}{\text{OD}520\text{ofcontrol}}\times 100 \end{array}$$

Where, the OD520 control and OD520 test are the absorbance of DMSO+ monitor strain and DMSO + monitor strain + crude extract, respectively.

#### Indirect assay for quorum-sensing inhibition using motility inhibition assay

The swarming and swimming motilities of *P. aeruginosa* MTCC2297 were analyzed using LB agar (0.5% W/V agar) plates amended with 100 μl (1 mg) of crude extract. The actively dividing cells of *P. aeruginosa* MTCC2297 were spot inoculated using a sterile wire stab at the center of the plate and incubated at 28°C for 24 h (Tremblay et al., 2007). DMSO alone was considered a negative control. The semisolid agar (0.3%) was supplemented with 100 μl (1 mg) of crude bacterial extract for swimming motility. The monitor strain was stabbed into the media between the bottom of the plate and the top of the agar (Zhang et al., 2018a). The motility of the cells through the surface of the agar or within the agar medium was observed, and the motility percentage was calculated using the following formula:


$$\begin{array}{l} \text{Motility inhibition \%}=\\ \frac{\text{Motility area of control}-\text{Motility area of the test}}{\text{Motility area of control}}\times 100 \end{array}$$

Where, motility area of control and motility area of the test are plates inoculated with DMSO and extracts, respectively.

### Evaluation of crude extracts for their anti-quorum-sensing potential against pathogen *L. amnigena*

#### Growth inhibition of *L. amnigena* RCE

The 100 μl of 24-h old (OD^600^; 0.4) culture of the pathogen was inoculated in the 10 ml LB broth supplemented with 100 μl (1 mg) of crude extracts and incubated at 28°C and 180 rpm in a shaking incubator. The DMSO supplementation was considered blank, and observation was recorded every 24 h intervals till 120 h.

#### Quantitative assay for biofilm inhibition of *L. amnigena* RCE

The 50 μl of 24 h old culture (OD^600^; 0.4) of *L. amnigena* RCE was added to the 5 ml LB broth containing 100 μl (1 mg) of crude extracts and incubated at 28°C. After incubation of 24 h, the tubes were washed with ddH_2_O and stained with 500 μl crystal violet (0.1%) for 30 min. The tubes were again washed with ddH_2_O, and cells were mixed with 2 ml of 95% ethanol (Passari et al., 2016; Zhang et al., 2018b). The ethanol phase was used to determine the violet color pigmentation reduction using UV-Vis spectrophotometer at 595 nm (UV-2600, Shimadzu, Japan). The DMSO was considered a negative control.


$$\begin{array}{l} \text{ Biofilm inhibition \%=}\\ \frac{\text{OD595 of control-OD595 of test}}{\text{OD595 of control}}\times \text{100} \end{array}$$

Where, OD595 control and OD595 test are the absorbance of DMSO and DMSO+ crude extract+ pathogen, respectively.

#### Maceration inhibition assay

The potato, carrot, and cucumber were purchased from the local market, and slices were made using a sterile scalpel. The slices were surface sterilized with 0.1% mercury chloride for 5 min and washed with sterile ddH_2_O. The slices were allowed to air dry under the aseptic inoculation chamber. The pathogen (*L. amnigena* RCE) and pathogen with bacterial extracts were evenly spread on the slice's center (100 μl and 200 μl). The DMSO, along with the pathogen, was considered a negative control. The slices were incubated at 28°C for 24 h under a sterile petri dish, and macerated tissue weight was measured by scooping out the macerated region (Garge and Nerurkar, 2016). Maceration (%) was calculated using the following formula:


$$\begin{array}{l} \text{ Maceration }(\text{\%})\text{=}\\ \frac{\text{macerated tissue weight of control macerated tissue weight of the test}}{\text{macerated tissue weight of control}}\times \text{100} \end{array}$$

Where control indicates pathogen with DMSO while test indicates pathogen and bacterial extracts.

#### Molecular identification of potential RB isolate

The DNA of bacteria was isolated by the LSP buffer method (Sheladiya et al., 2022). The 16S rRNA gene-specific primer; 27F 5‘AGAGTTTGATCCTGGCTCAG-3' and 1492R 5‘GGTTACCTTGTTACGACTT 3' were used to amplify the target sequence of 1,500 bp. The amplified product was subjected to sequencing at SLS research Pvt. Ltd., Surat, India. The sequence result was aligned using the nBLAST tool of NCBI (National Center for Biotechnology Information). The sequence was submitted to the NCBI through the BankIt submission system to obtain a unique accession number (https://www.ncbi.nlm.nih.gov). The organisms showing more than 99% similarity were considered for constructing the phylogenetic tree using the MEGA 7 tool (Kumar et al., 2016).

### Identification of QSI metabolites by GC-MS

#### Analysis of metabolites through well diffusion assay

The 24-h-old culture of *L. amnigena* was spread evenly on LA plates, and wells were formed using an 8 mm sterile borer. Hundred microliter of crude extract was loaded into the wells. The plates were incubated at 28°C for 24 h. The clear zone around the well was taken into a separate glass vial using a sterile scalpel and dipped in 5 ml of GC-grade acetonitrile (Sigma-Aldrich, USA). The samples were vortexed, centrifuged, and filtered for GC-MS analysis. The metabolites diffused through the agar and in the clear zone were analyzed. The dry bacterial extract was mixed with GC-grade acetonitrile and analyzed through GC-MS.

#### Extraction of metabolites from liquid culture

The 100 μl overnight grown culture of *L. amnigena* RCE was added to the 10 ml LB media supplemented with 100 μl (1 mg) of crude extracts. The tubes were incubated at 28°C from 24 h to 96 h at 180 rpm. The bacterial suspension was collected every 24 h interval till 96 h and centrifuged. The supernatant was mixed with an equal volume of ethyl acetate and dried under a vacuum evaporator. The dry extract was mixed with 1.0 ml GC grade acetonitrile (Sigma-Aldrich, USA).

#### Characterization of metabolites by GC-MS

The final extract was transferred into screw neck GC glass vial with cap and PTFE/silicone septa after filtration through 0.22 μm syringe filter to remove cell debris and preventing the accumulation of fine particles that can block chromatographic column. The chromatographic analysis was performed on Thermo Scientific made triple quadrupole GC-MS/MS system (Model: TSQ 9000) with Advanced Electron Ionization (AEI) Source. The Gas chromatograph (Model: TRACE 1300) was attached to MS system for chromatographic separation. Helium gas (purity; 99.999%) was used as carrier gas in constant flow mode (1.0 ml/min). The capillary column Restek™ Rxi-5 MS (0.25 um Thickness; 0.25 mm ID; 30 m Length) was used. The injection was done using autosampler (AI 1310). The sample was injected in split less mode while injector temperature was kept at 280°C. The temperature of transfer line and ion source temperature were set at 280 and 310°C, respectively. The oven temperature program used for separation was as follows: The initial oven temperature was kept at 90°C for 5 min, then increased at rate of 25°C/min till 180°C, then it was increased from 180 to 280°C at a rate of 5°C/min, lastly it ramped with 10°C/min until it reached 300°C followed by a hold for 1.4 minute. The total run time was 32.0 min. The Thermo Scientific™ Chromeleon™ 7.2 CDS (chromatography data system) used. The full scan mode (*m/z* 50–550) was used to detect the metabolites. The identification of the metabolites was done by comparing the ions detected in the scan to ions recorded in the built library i.e., The NIST (National Institute for Standard and Technology library) 2.0 Mass Spectral Library.

#### Statistical analysis

The mean and standard deviation was used to deduce the conclusive results of the experiment. The Metabo Analyst 5.0 (Pang et al., 2021) was used to analyze the complex metabolic pattern in each sample. The spectra and ions were matched with the known spectra present in the NIST library to get the probable name of the metabolites distinctly present in the samples. The data were first normalized and assigned common names while areas of similar chemicals were merged. The principal component analysis (PCA), partial least square discriminate analysis (PLS-DA), a heat map, and other analyses were carried out to generate diverse results (Pang et al., 2021). Venny 2.1 online tool was used to identify the common and distinct metabolites present in different samples (Oliveros, J. C. (2007–2015) (https://bioinfogp.cnb.csic.es/tools/venny/index.html).

## Results

### Isolation of bacteria

**Figure 1:**
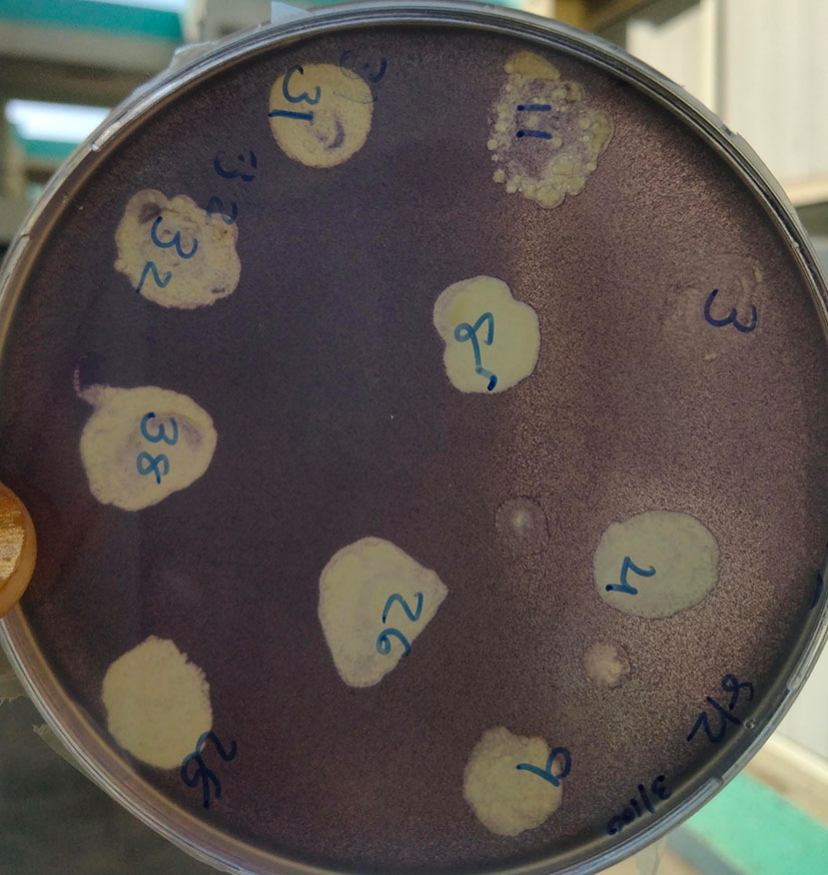
Isolation and screening of quorum-sensing inhibiting bacterial isolates.

There were 59 isolates which showed purple discoloration of monitor strain *C. violaceum* MTCC2656. The positive isolates (Figure 1) were sub-cultured repeatedly to get a pure uniform colony.

### Well diffusion assay of crude extract

**Figure 2:**
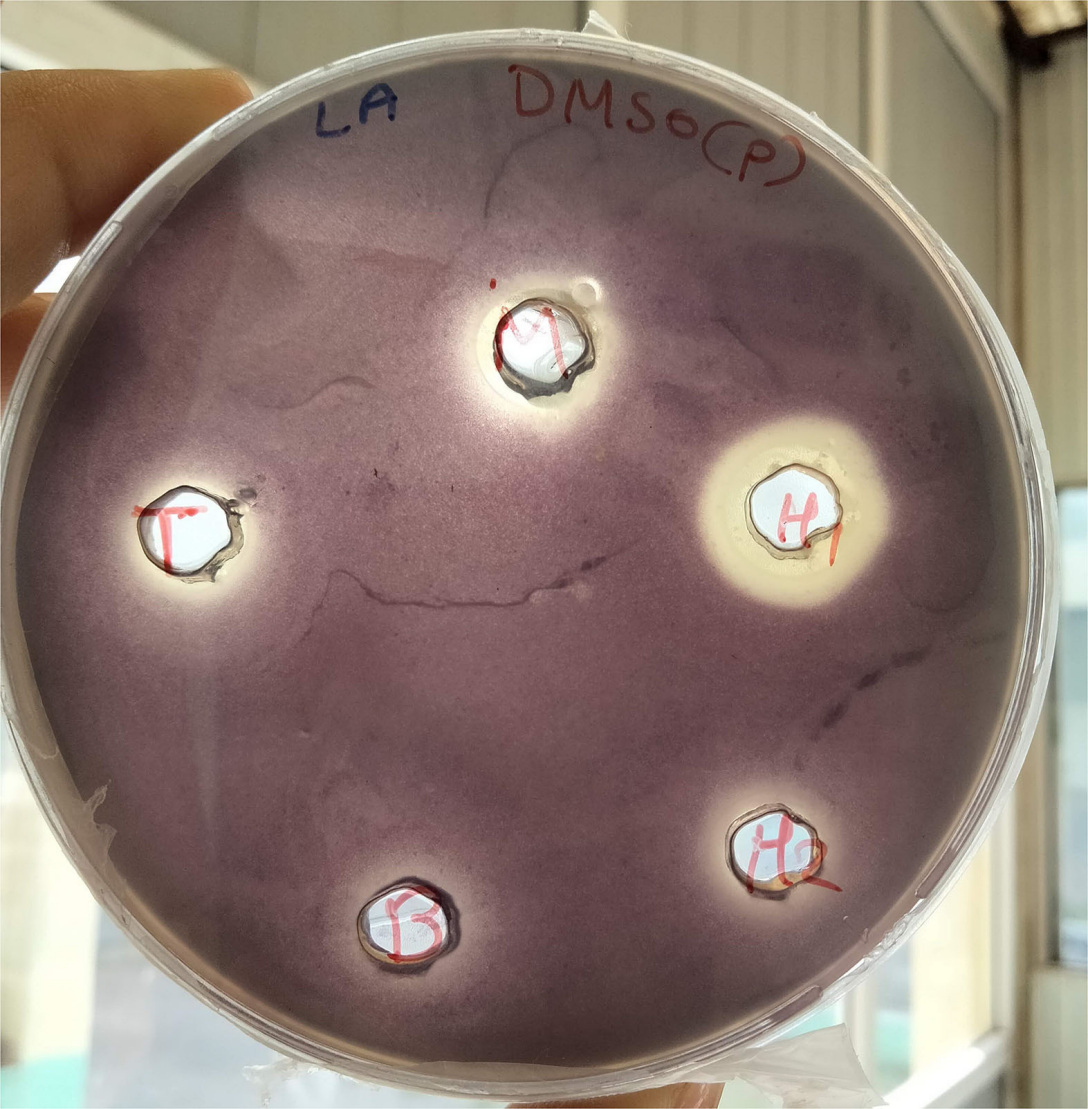
Well diffusion assay of bacterial crude extract.

Among the potent isolates, five isolates were used to extract metabolites using ethyl acetate. The isolate, RB, exhibited 12 mm of purple discoloration on well diffusion agar plate assay without affecting the growth of monitor strain; therefore, the extract was further studied (Figure 2). The concentration and different nature of compounds may be the reason for varied pigment inhibition by the extract. The diverse organisms might exhibit several strategies to survive in the environment and subsequently either inhibit or assist the growth of other microbes.

### Violacein and pyocyanin inhibition assay

**Figure 3:**
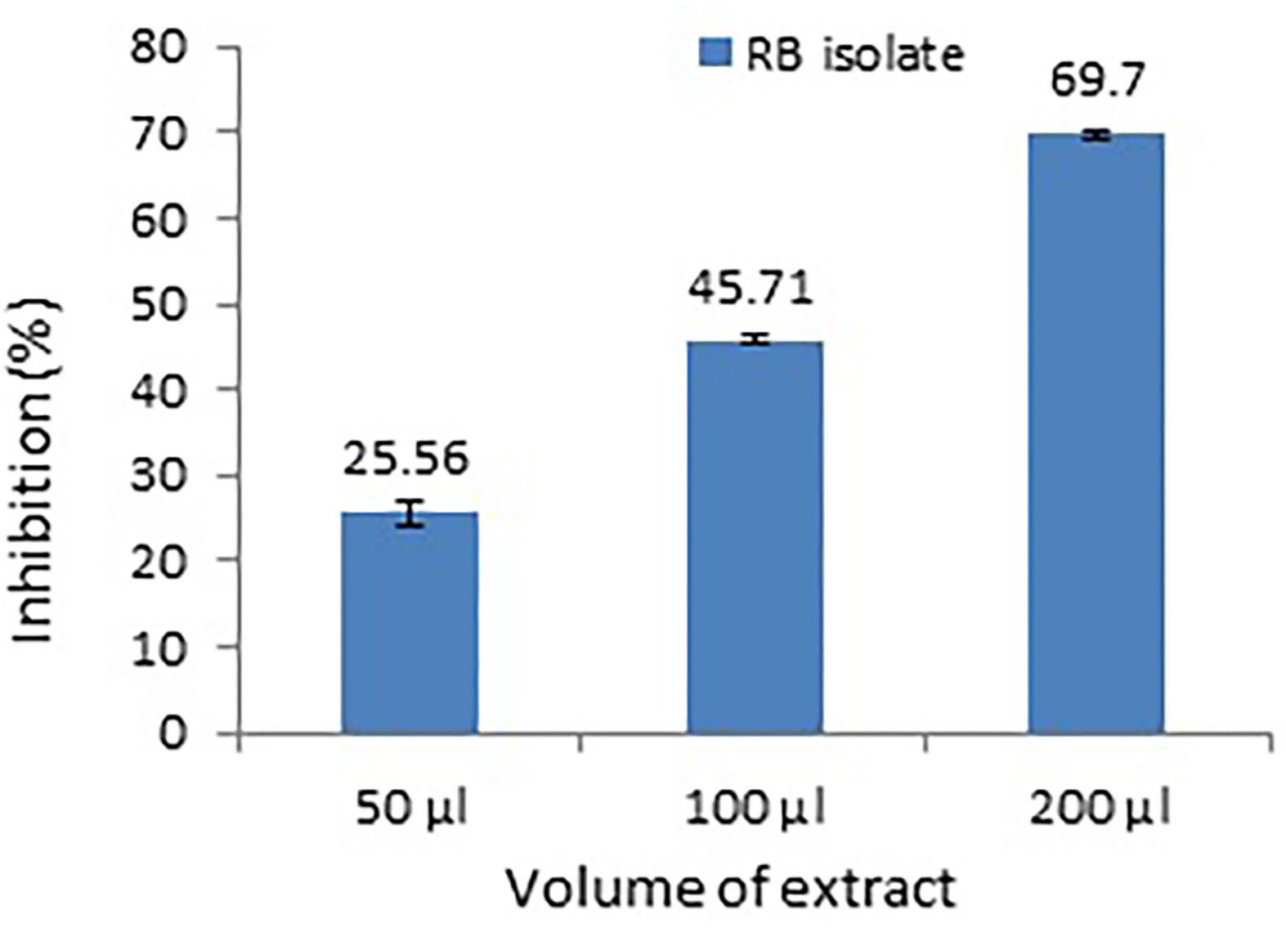
Dose-dependent inhibition of violacein production in a monitor strain, *C. violaceum* MTCC2656. Values represent the mean of three replications. Bars indicate the standard error of the mean.

**Figure 4:**
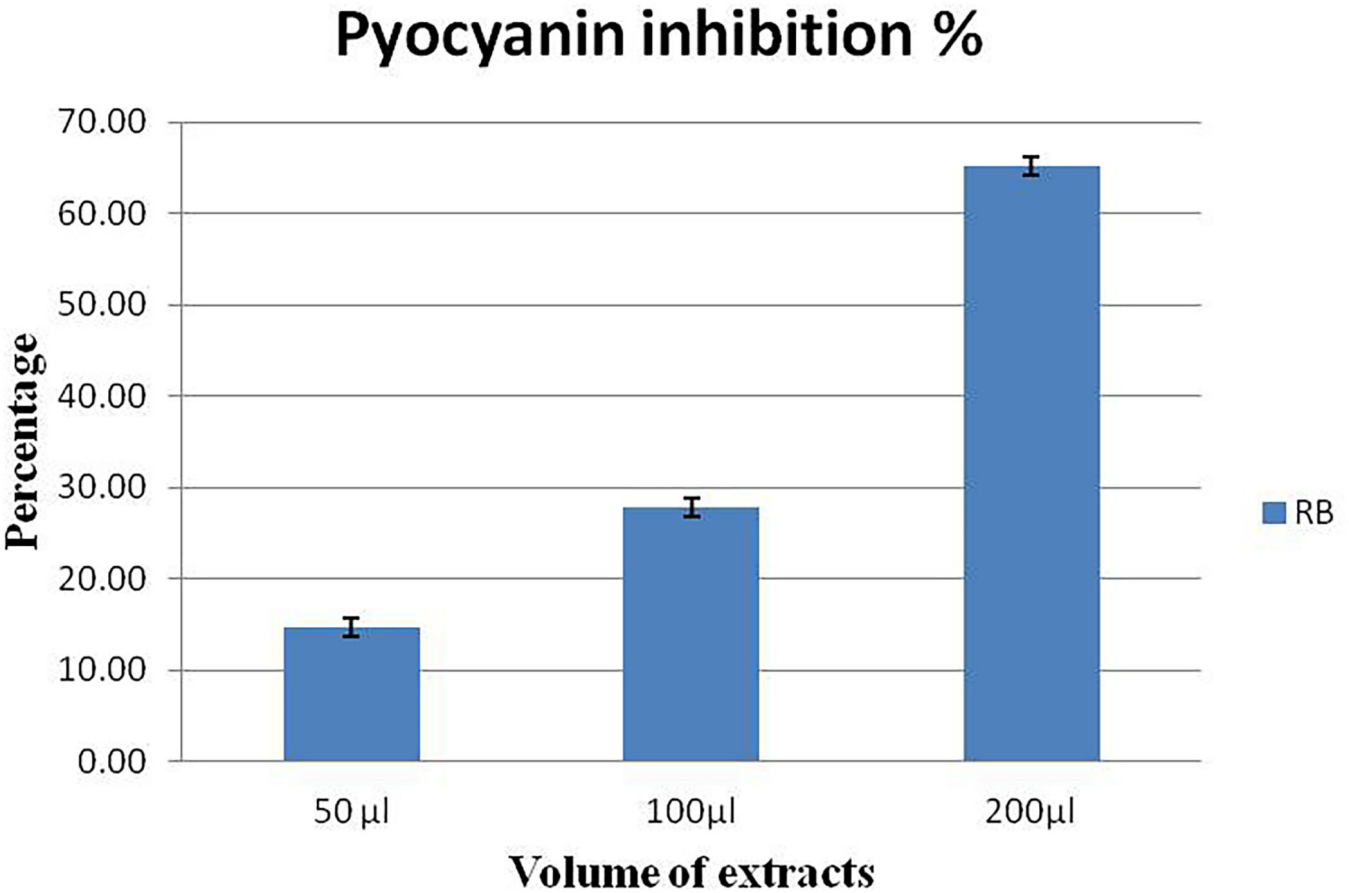
Concentration-dependent inhibition of pyocyanin pigment in *P. aeruginosa* MTCC2296. Values represent the mean of three replications. Bars indicate the standard error of the mean.

*P. aeruginosa* MTCC2297 has been widely employed to determine quorum-sensing inhibition properties of several bacterial extracts. During the initial assay, 50 μl (10 mg/ml) gave better results. Hence, concentration-dependent inhibition of pigment production in *C. violaceum* MTCC2656 was monitored by increasing the concentration of extracts. The inhibition percentage of violacein pigment is depicted in a bar graph along with their mean (Figure 3). During the quantitative assay, the higher RB bacterial crude extract concentration inhibited the violacein pigments up to 69.7%. The RB extracts also noticed the dose-dependent inhibition of pyocyanin, and it was reported to be 65.21% (Figure 4).

### Swimming and swarming motility assay

**Figure 5:**
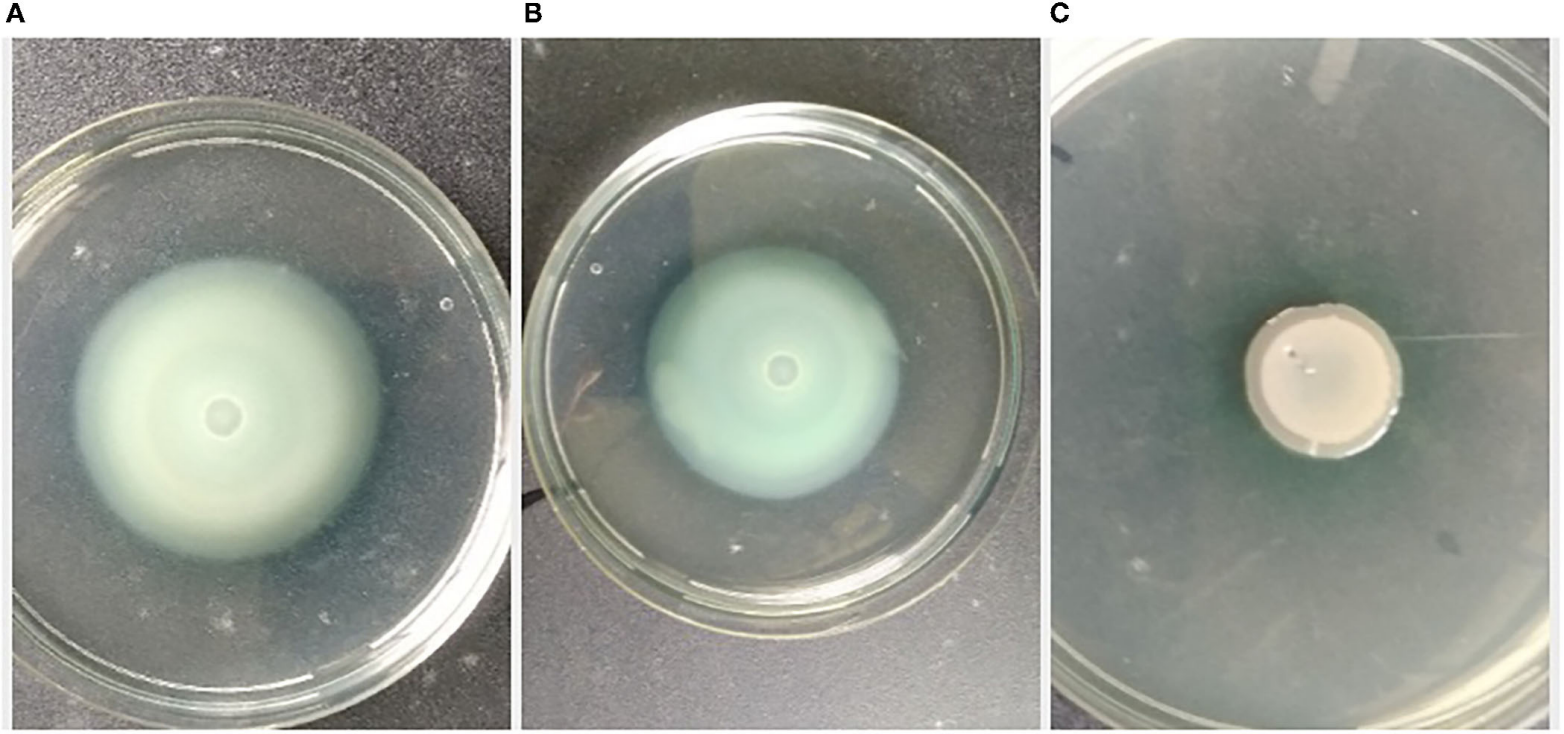
Effect of crude extract on swimming motility of *P. aeruginosa* MTCC2297; **(A)** *P. aeruginosa* 2,297 alone, **(B)** *P. aeruginosa* 2,297 + DMSO (Control), and **(C)** Ethyl acetate extract of RB isolates (100 μl).

**Figure 6:**
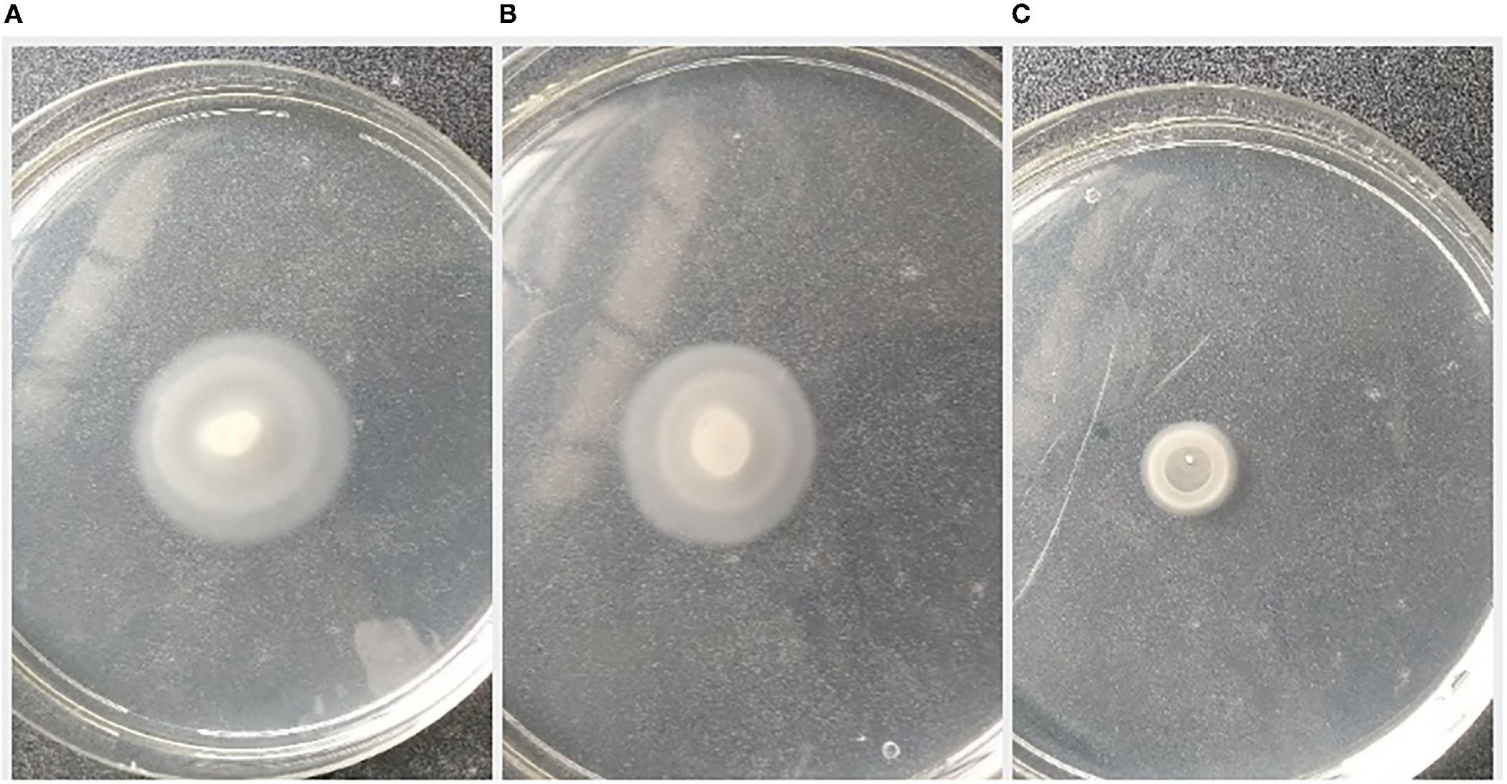
Effect of crude extract on swarming motility of *Pseudomonas aeruginosa* MTCC2297; **(A)** *P. aeruginosa* 2,297 alone, **(B)** *P. aeruginosa* 2,297 + DMSO (Control), and **(C)** Ethyl acetate extract of RB isolate (200 μl).

The pathogen, *L. amnigena* strain RCE (MZ712952), contains peritrichous flagella, which are responsible for their movement. The indirect assay was performed using *P. aeruginosa* MTCC2297, and motility inhibition was monitored (Figure 5). The RB extract reduced the swimming and swarming motility by 51.57 and 56.80%, respectively (Figures 5, 6). These results substantiate RB bacterial extract's potential for anti-quorum sensing.

### Quantification of biofilm inhibition and antibacterial assay

**Figure 7:**
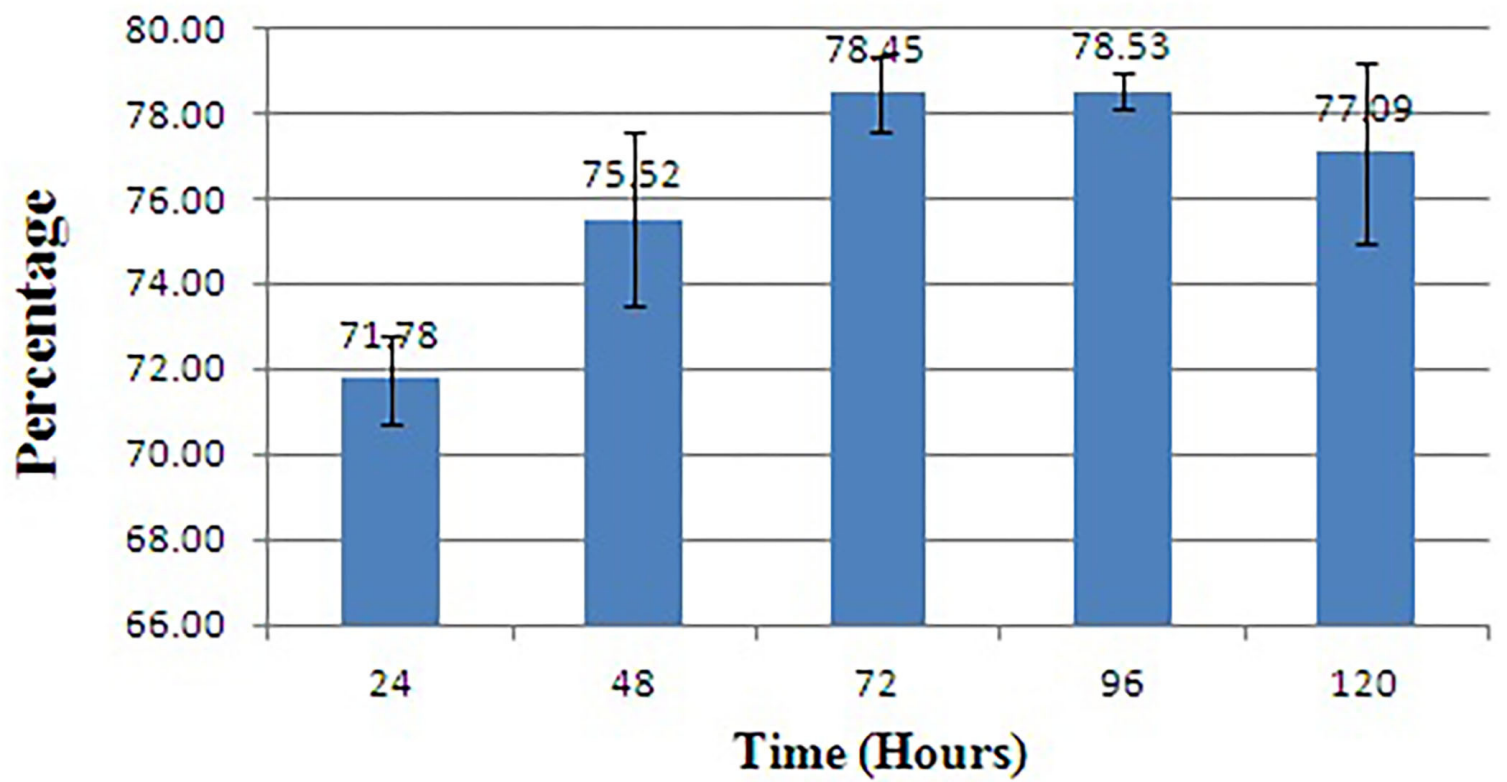
Biofilm inhibition of *L. amnigena* RCE. Values represent the mean of three replications. Bars indicate the standard error of the mean.

**Figure 8:**
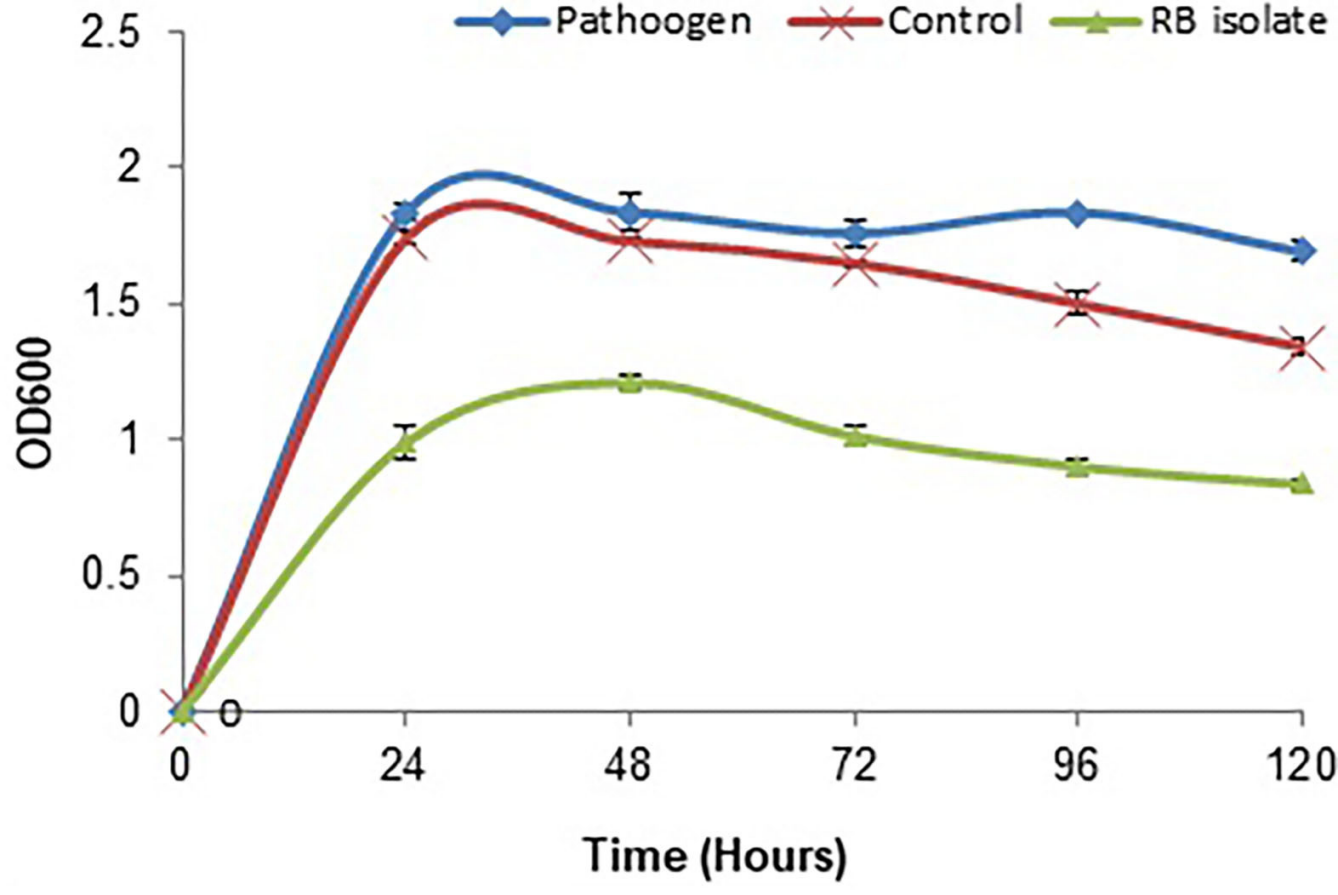
Growth inhibition of *L. amnigena* RCE by crude extract of bacteria. Values represent the mean of three replications. Bars indicate the standard error of the mean.

The biofilm is considered one of the potent virulence factors of the Gram-negative bacterium *L. amnigena* RCE. Supplementation of RB extracts to the pathogen reduces biofilm formation up to 78% even after incubation for 120 h (Figure 7). The growth curve of *L. amnigena* RCE alone or with DMSO (control) in the Luria broth showed similarity in their growth pattern while supplementation of crude extracts reduced the growth at the initial period of 24 h than after the growth was increased a little bit till 48 h followed by decreases further (Figure 8). This indicated that extracts might have some components that initially inhibit pathogen growth compared to control, but pathogen overcomes such effects by their metabolisms.

### *In vitro* soft rot attenuation on different host plant

**Figure 9:**
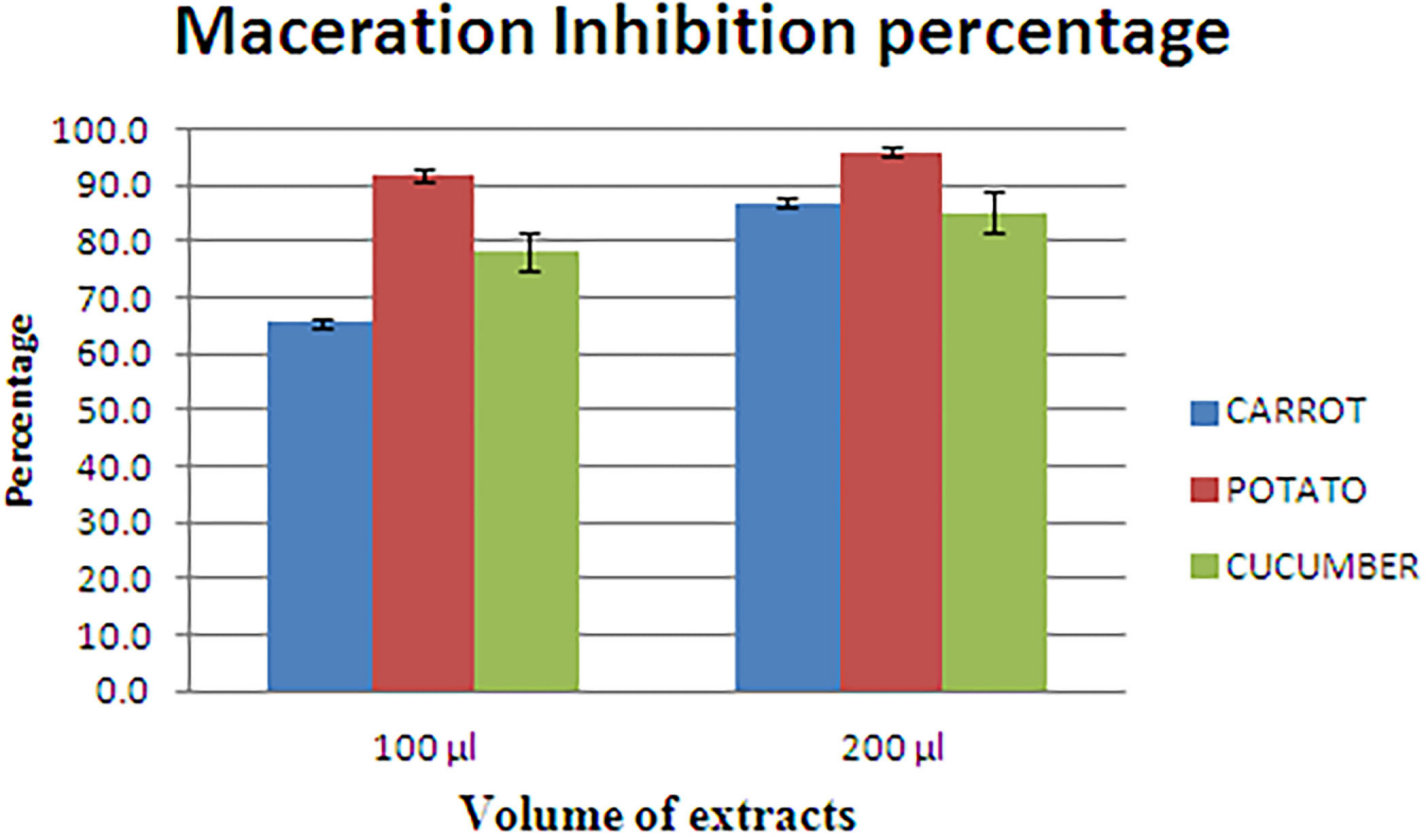
Maceration inhibition assay of potato, carrot, and cucumber using RB extract.

**Figure 10:**
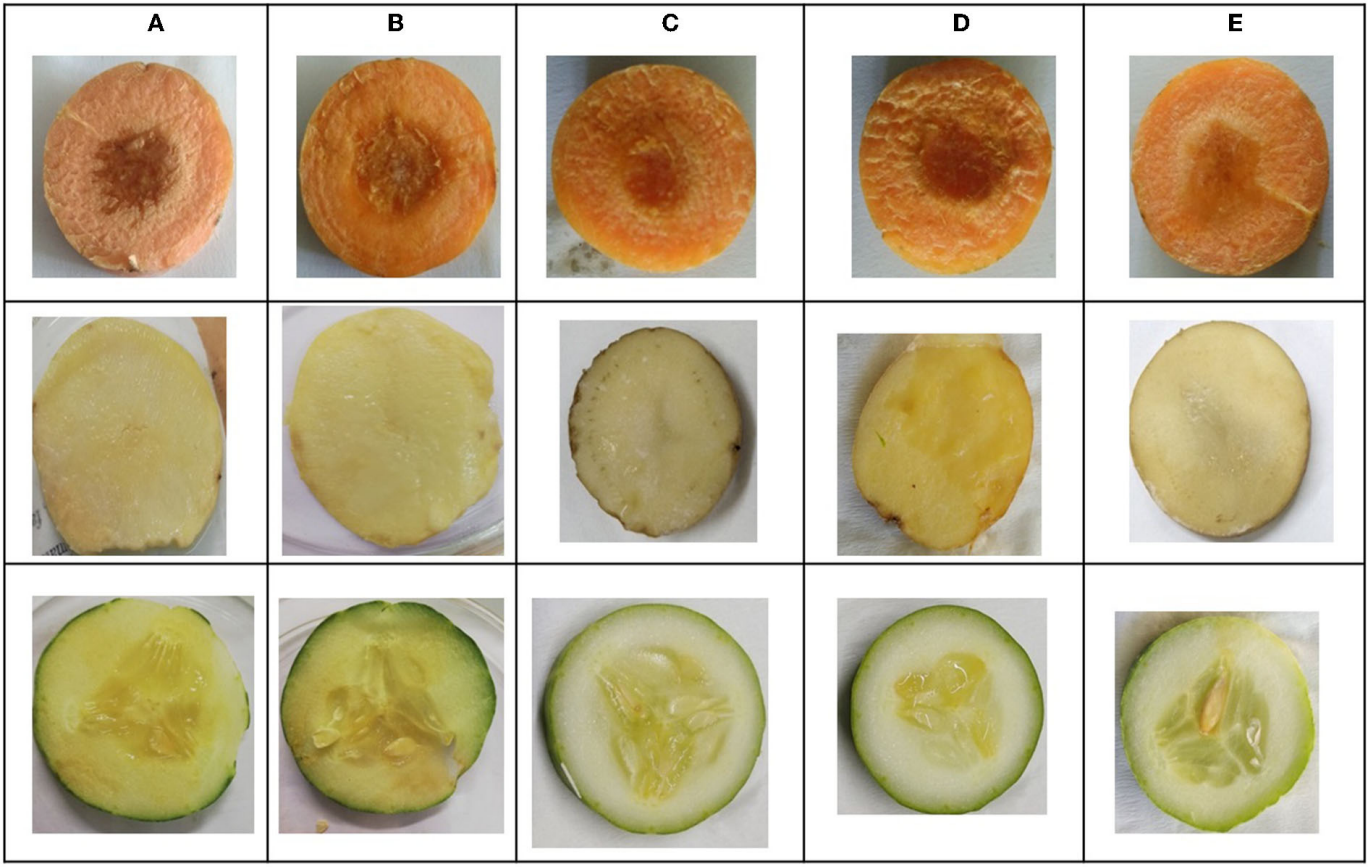
*In vitro* soft rot attenuation assay on potato, carrot, and cucumber; **(A)** *Lelliottia amnigena* alone, **(B)** *L. amnigena* RCE + DMSO (Control, 100 μl), **(C)** RB bacterial crude extract (100 μl) + *L. amnigena* RCE (100 μl), **(D)** *L. amnigena* RCE + DMSO (Control, 200 μl), and **(E)** RB bacterial crude extract (200 μl) + *L. amnigena* RCE.

*L. amnigena* RCE is the potent soft rotting pathogen and secretes several pectate cell wall degrading enzymes. The secretion of enzymes is mediated by the quorum-sensing signal molecules of the pathogen. When potato, carrot, and cucumber slices treated with *L. amnigena* RCE alone or co-inoculation with RB bacterial extract, the soft rot symptoms were appeared to be reduced in a concentration-dependent manner by 95.95, 86.99, and 85.25%, respectively (Figure 9). DMSO with pathogen and pathogen alone treatments were considered as controls, showing a higher maceration percentage (Figure 10).

### Molecular identification and phylogenetic analysis

The 16s rRNA gene sequence of the RB isolates was amplified and sequenced. The RB isolate showed 96.74% identity with *P. aeruginosa* strain DBT20 (MF421779.1). The 16s rRNA gene sequence was submitted to the genebank database under the accession number MZ068216.1. The isolate had higher sequence similarity with the respective reference strain in the database; hence RB isolate was initially named *P. aeruginosa* RKC1 (Supplementary Figure S1).

### GC-MS analysis

**Figure 11:**
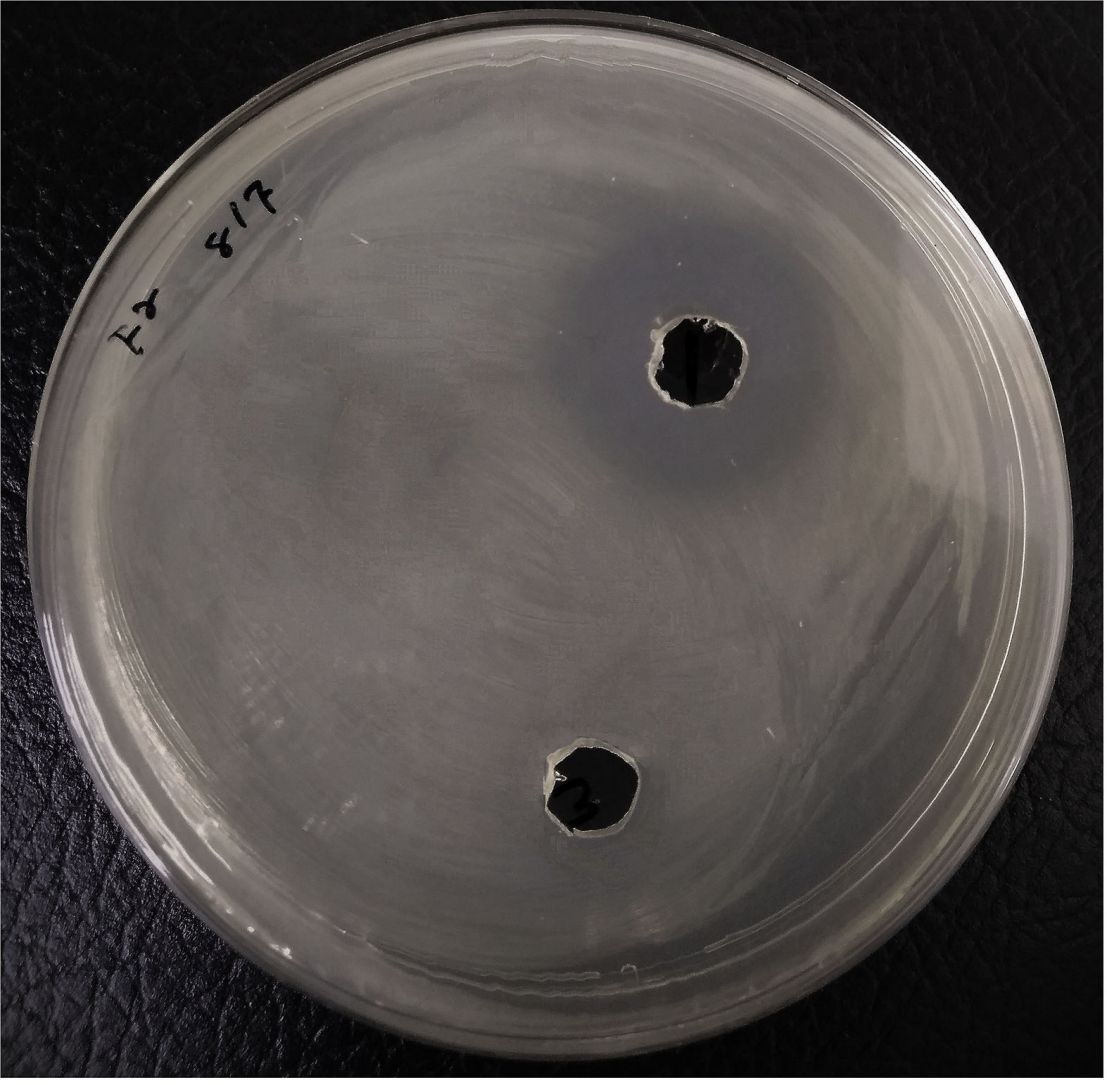
Agar well diffusion assay using bacterial extracts loaded in the wells and *Lelliottia amnigena* RCE spread over the agar. Control has the same concentration of DMSO.

**Figure 12:**
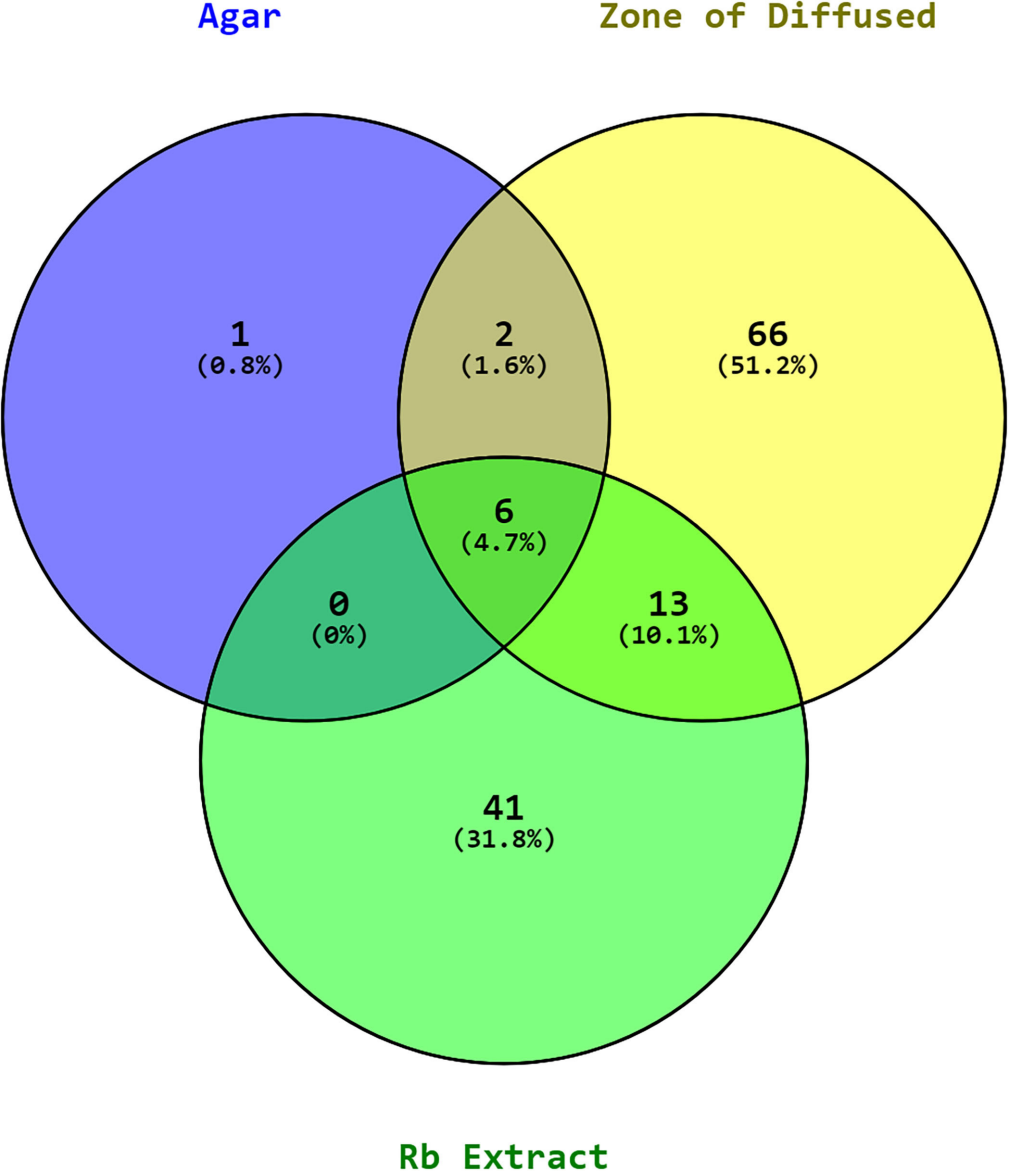
Venn diagram of metabolites in the RB (*P. aeruginosa* RKC1) extract, the clear zone around the well, and agar.

The experiments were carried out using the ethyl acetate extracts of *P. aeruginosa* RKC1, which showed quorum-sensing inhibition activities against monitor strain and pathogen *L. amnigena* RCE. To understand the interaction of metabolites and their resultant effects, the well diffusion assay was conducted, and the metabolic profile of extracts was analyzed using GC-MS. Moreover, molecules that travel well through the solid medium were also characterized using GC-MS (Figure 11). The Venn diagram is the easiest way to classify the distinguished metabolites in different samples. As per the Venn diagram, 66 metabolites were found to be diffused from the wells through the solid agar toward the pathogen or may be released by the pathogen, while 41 metabolites were exclusively present in the extract. There were 13 common metabolites (Table 1) found in the clear zone around the well and bacterial extracts (Figure 12). The diketopiperazines are predominantly found in extracts and a clear zone around the well, which accounts for 9.66% of the peak area.

**Table 1:** List of metabolites obtained through GC-MS.

**A**	**B**
Benzamide	Gancidin W or **Cyclo (L-Leu-L-Pro)** or pyrrolopyrazine
3-Methyl-2,3,6,7,8,8a-hexahydropyrrolo[1,2-a]pyrazine-1,4-dione, or **Cyclo (Pro-Ala)**	Cyclo **(prolyl tyrosyl)**
Hexadecanal	7,9-Di-tert-butyl-1-oxaspiro(4,5)deca-6,9-diene-2,8-dione or Lectone or cyclic ketone
**Cyclo (L-prolyl-L-valine),**	cis-11-Eicosenamide
Pyrrolo[1,2-a]pyrazine-1,4-dione, hexahydro-3-(2-methylpropyl)-, or **Cyclo (Pro-Leu)**	Glycyl-L-proline
2,8,9-Trioxa-5-aza-1-silabicyclo[3.3.3]undecane,1-ethenyl-,	**Cyclo-Ala-Pro-diketopiperazine**
Octadecanoic acid,	**Cyclo L–prolyl–L–val**
Glycidyl palmitate,	**Cyclo–Trp–Pro**
Pyrrolo[1,2-a]pyrazine-1,4-dione, hexahydro-3-(phenylmethyl)-, or **Cyclo (Phe-Pro)**	2-(2-Oxo-2,3-dihydro-1H-imidazol-4-yl)malonic acid, diethyl ester
4-Hexyl-1-(7-methoxycarbonylheptyl)bicyclo[4.4.0]deca-2,5,7-triene,	Palmitoyl chloride or Hexadecanoic acid chloride
(E)-4-Hydroxy-4-[4-hydroxy-2-[(E)-6-hydroxyhept-1-enyl]cyclopentyl]but-2-enoic acid, 4Me derivative (Hydroxy fatty acid)	D–Mannose
cis-11-Eicosenamide	Hexadecanoic acid
Squalene	6-Benzyl-3-methyl-6,7-dihydro-4H-isoxazolo[5,4-c]pyridin-5-one
	Oxychlororaphine
	7-Ethyl-4,6-pentadecandione
	**Cyclo Leu–Ala**
	**Cyclo_Phe-Val_**

*A. Metabolites found common in extract of Pseudomonas aeruginosa RKC1 and clear zone around the well, B. Metabolites found in high concentration in P. aeruginosa RKC1 extract*.

Moreover, the PLS-DA analysis of the data provides important features to draw valuable conclusions by considering VIP scores >1.5 (Supplementary Figures S2, S3). The Palmitoyl chloride, 1, 6-Dioxacyclododecane-7, 12-dione, n–Hexadecanoic acid, and 1-Cyclohexyldimethylsilyloxy-3,5-dimethyl benzene were shown to have higher peak area that indicated their secretion by the pathogen and diffused through the agar. To further confirm the hypothesis, the experiment was set up to identify differentially regulated metabolites of the pathogen at different times during interaction with *P. aeruginosa* RKC1 extract. Some metabolites might be present in the extract that represses certain metabolites released by the pathogen during its active growth in media (Table 2). The cyclic dipeptides, also known as 2,5-diketopiperazines (DKPs) of different types, were largely present by almost 93.72% of the total peak area in the extract of *P. aeruginosa* RKC1 (Table 3). These metabolites might have a key role in the quorum quenching of pathogen. The peak areas of some DKPs are very less than the extracts, and even some were not detected at all, which their lower mobility could explain through the agar (Table 4). To deduce the relevance of such metabolites during the active growth phase, the ethyl acetate extracts of bacteria were added to the pathogen, and metabolite profiling was done using GC-MS at an interval of every 24 h (Supplementary Figures S4, S5). There were various metabolites, as listed in Table 2, which were produced by the pathogen during their active growth. In contrast, the interaction of the pathogen with metabolite showed downregulation of all such metabolites, which could be inferred from the heat map and their peak area percentages (Table 3). The Gancidin W or Cyclo (L-Leu-L-Pro), 3-Benzylidene-hexahydro-pyrrolo_1_2-a_pyrazin-1_4-dione, 5-Formyluracil, Benzoic acid, 4-[(trimethylsilyl) oxy]-, phenyl ester, and 12-Hydroxy-14-methyl-oxa-cyclotetradec-6-en-2-one were considered to be an important parameter obtained through statistical analysis (Supplementary Figure S6).

**Table 2:** Metabolites regulated during active growth of pathogen.

Regulated and Produced by pathogen and regulated during interaction with bacterial extract
Squalene
Tyrosol
Tryptophol
4-(4-tert-butylphenyl)-1,3-thiazol-2-amine
Benzoic acid, 4-[(trimethylsilyl)oxy]-, phenyl ester
1–Octylsilatrane
4-Tetradecylmorpholine
Ethyl 4-ethoxybenzoate
Ethyne, bis(dicyclohexylphosphino)-
1,6-Dioxacyclododecane-7,12-dione
2-Resorcylic_acid
Octadecanal
2-oxo-3-phenylpropanal
Dodecanal
2-Hydroxy-3-methoxybenzaldehyde
Ethyl 4-ethoxybenzoate
Hexahydro-3-(1-methylpropyl)pyrrolo[1,2-a]pyrazine-1,4-dione
3-Benzylidene-hexahydro-pyrrolo_1_2-a_pyrazin-1_4-dione
2,4-dimethylbenzo[h]quinoline
2,5-Piperazinedione, 3-benzyl-6-isopropyl-
2,4 DTBP
5-Formyluracil
1-Cyclohexyldimethylsilyloxy-3_5-dimethylbenzene

**Table 3:** Metabolite present in the ethyl acetate extract of *Pseudomonas aeruginosa* RKC1.

**Metabolites present in the ethyl acetate extract of *Pseudomonas aeruginosa* RKC1**	**Area**	**%** **(Total area)**	**RT (Min.)**
Benzamide	398,241	0.05	8.63
d-Mannose	2,128,503	0.28	8.84
4-(4-tert-butylphenyl)-1,3-thiazol-2-amine	409,292	0.05	11.05
2,4-dimethylbenzo[h]quinoline	4,756,787	0.62	11.76
**Cyclo-Ala-Pro-diketopiperazine**	3,910,039	**0.51**	11.84
**Cyclo (Leu-Ala)**	2,233,361	**0.29**	11.93
Tetradecanoic acid	633,341	0.08	12.19
**N-glycylproline**	2,491,758	**0.32**	12.28
Tryptophol	767,113	0.10	12.41
Benzoic acid, 4-[(trimethylsilyl)oxy]-, phenyl ester	371,851	0.05	12.69
Octadecanal	341,507	0.04	12.90
**Cyclo (L-prolyl-L-valine)**	430,824,824	**56.06**	13.03
Benzoic acid, 4-[(trimethylsilyl)oxy]-, phenyl ester	2,440,928	0.32	13.12
7-Ethyl-4,6-pentadecandione	3,795,209	0.49	13.26
4-Tetradecylmorpholine	677,619	0.09	13.74
**Hexahydro-3-(1-methylpropyl)pyrrolo[1,2-a]pyrazine-1,4-dione or Cyclo (L-prolyl-L-valine)**	31,818,750	**4.14**	14.22
7,9-Di-tert-butyl-1-oxaspiro(4,5)deca-6,9-diene-2,8-dione	3,936,789	0.51	14.33
**Pyrrolo[1,2-a]pyrazine-1,4-dione, hexahydro-3-(2-methylpropyl)- or Cyclo (Pro-Leu)**	142,024,854	**18.48**	14.44
n-Hexadecanoic acid	7,562,556	0.98	14.65
2-(2-Oxo-2,3-dihydro-1H-imidazol-4-yl)malonic acid, diethyl ester	925,371	0.12	15.11
2,5-Piperazinedione, 3-methyl-6-(1-methylpropyl)-	221,222	0.03	15.46
1-Octylsilatrane	883,001	0.11	17.20
Palmitoyl chloride	3,267,934	0.43	17.41
Pent-4-enoylamide, 2-methyl-N-(2-butyl)-N-pentyl-	1,402,868	0.18	18.48
2,5-Piperazinedione, 3-benzyl-6-isopropyl-	793,667	0.10	19.09
1-Cyclohexyldimethylsilyloxy-3,5-dimethylbenzene	3,981,245	0.52	20.37
**Pyrrolo[1,2-a]pyrazine-1,4-dione, hexahydro-3-(phenylmethyl)- or Cyclo(D-phenylalanyl-L-prolyl)**	96,911,660	**12.61**	20.90
6-Benzyl-3-methyl-6,7-dihydro-4H-isoxazolo[5,4-c]pyridin-5-one	5,287,689	0.69	21.05
Phenazine-1-carboxamide	2,097,106	0.27	21.22
**Cyclo(prolyl tyrosyl)**	8,238,058	**1.07**	25.86
1,3-Benzenedicarboxylic acid, bis(2-ethylhexyl) ester	472,626	0.06	26.09
cis-11-Eicosenamide	532,888	0.07	26.52
Squalene	210,168	0.03	27.22
**cyclo-(L-Trp-L-Pro)**	1,741,648	**0.23**	30.84

**Table 4:** Metabolite present in the clear zone around the well (Zone of diffused metabolites).

**Metabolites present in the clear zone around the wells (Well diffusion assay)**	**Area**	**%** **(Total area)**	**RT (Min.)**
2-oxo-3-phenylpropanal	242,741	0.77	8.172
Benzamide	750,189	2.39	8.628
Dodecanal	576,252	1.84	9.175
2-Hydroxy-3-methoxybenzaldehyde	283,600	0.91	9.654
2,4-DTBP	124,152	0.40	9.983
Ethyl 4-ethoxybenzoate	153,263	0.49	10.117
5-Formyluracil	134,212	0.43	10.181
4-Deoxypyridoxine	82,859	0.26	10.305
1,6-Dioxacyclododecane-7,12-dione	840,414	2.68	10.395
n-tetradecyl methyl imine	179,584	0.57	10.55
4-(4-tert-butylphenyl)-1,3-thiazol-2-amine	124,535	0.40	11.063
2-Quinolinylmethanol	83,967	0.27	11.532
2,4-dimethylbenzo[h]quinoline	125,851	0.40	11.744
Tetradecanoic acid	165,635	0.53	12.196
**N-glycyl proline**	1,839,883	**5.87**	12.274
Tryptophol	217,035	0.69	12.455
Benzoic acid, 4-[(trimethylsilyl)oxy]-, phenyl ester	314,894	1.01	12.679
Octadecanal	666,672	2.13	12.901
**Hexahydro-3-(1-methylpropyl)pyrrolo[1,2-a]pyrazine-1,4-dione or Cyclo(L-prolyl-L-valine)**	320,029	**1.02**	13.015
Benzoic acid, 4-[(trimethylsilyl)oxy]-, phenyl ester	542,157	1.73	13.122
7-Ethyl-4,6-pentadecandione	54,365	0.17	13.226
4-Tetradecylmorpholine	239,361	0.76	13.766
7,9-Di-tert-butyl-1-oxaspiro(4,5)deca-6,9-diene-2,8-dione	324,248	1.04	14.312
**Pyrrolo[1,2-a]pyrazine-1,4-dione, hexahydro-3-(2-methylpropyl)- or Cyclo (Pro-Leu)**	353,120	**1.13**	14.406
n-Hexadecanoic acid	7,911,992	25.26	14.651
2-(2-Oxo-2,3-dihydro-1H-imidazol-4-yl)malonic acid, diethyl ester	170,808	0.55	15.127
1-Octylsilatrane	762,312	2.43	17.22
Palmitoyl chloride	3,140,462	10.02	17.421
Pent-4-enoylamide, 2-methyl-N-(2-butyl)-N-pentyl-	826,097	2.64	18.488
1-Cyclohexyldimethylsilyloxy-3,5-dimethylbenzene	2,101,607	6.71	20.433
**Pyrrolo[1,2-a]pyrazine-1,4-dione, hexahydro-3-(phenylmethyl)- or Cyclo(D-phenylalanyl-L-prolyl)**	512,508	**1.64**	20.849
6-Benzyl-3-methyl-6,7-dihydro-4H-isoxazolo[5,4-c]pyridin-5-one	619,069	1.98	21.073
3-Benzylidene-hexahydro-pyrrolo[1,2-a]pyrazin-1,4-dione	4,207,362	13.43	22.512
1,3-Benzenedicarboxylic acid, bis(2-ethylhexyl) ester	462,921	1.48	26.08
cis-11-Eicosenamide	524,558	1.67	26.52
Squalene	317,940	1.01	27.217
Phenol, 2,4-bis(1,1-dimethylethyl)-, phosphite (3:1)	1,029,805	3.29	28.27

## Discussion

Microorganisms communicate with each other through the secretion of diverse classes of signal molecules. The accumulation of signal molecules above their threshold in the microbes' environments leads to regulating several genes (Abisado et al., 2018). These signal molecules freely diffuse to the cells and bind to their respective receptors, thus regulating various pathogenesis-related traits. The production of EPS (Ilyas et al., 2020; Sheikh et al., 2022), pigment (Polapally et al., 2022), toxins, formation of biofilm (Ali et al., 2022; Fazeli-Nasab et al., 2022), and secretion of cell wall degrading enzymes (Jadhav et al., 2020) are key traits expressed by the pathogen once sufficient cell density is achieved (Sibanda et al., 2018). This quorum-sensing guided virulence factor synthesis can be controlled by disturbing the QS system either by mimicking the signal molecules, which leads to competitive inhibition, or enzymatically hydrolyse the auto-inducers (N-Homoserine Lactone). In conventional approaches, various biocontrol (Vinay et al., 2016; Reshma et al., 2018; Kusale et al., 2021; Mulk et al., 2021; Nakkeeran et al., 2021; Nithyapriya et al., 2021; Sukmawati et al., 2021; Khan, 2022) substances are used to control pathogens, but repeated use of such molecules lead to the development of multidrug-resistant bacteria which would be very difficult to eradicate further.

The desperate attempts require to identify novel antimicrobial agents or developing new procedures to thwart antibiotic resistance (Abdalla et al., 2020; Kapadia et al., 2021). The advanced and innovative antimicrobial agents developed so far target only the most prominent pathogenic bacteria (Dellit et al., 2007), while less severe pathogenic bacteria remain resistant (Masood et al., 2021; Mubraiz et al., 2021). Therefore, developing innovative strategies which target pathogenesis-responsible compounds are of prime importance for controlling pathogen. The actinobacteria produce several classes of bioactive metabolites which has the potential to act as anti-biofilm agents against several pathogens (Deka et al., 2020). The virulence in the pathogen is governed by the coordinated regulation of synthesis, accumulation, transport, and receptor molecules. The hydrolysis, transformation of signal molecules, or blockage of synthase and receptor lead to disturbance in the communication system of the cells (LaSarre and Federle, 2013). For any quorum-sensing guided system, synthase produces diverse types of signal molecules which freely move across the membranes and accumulate in cell surroundings. At the same time, its cognate receptors sense the signal molecules resulting in the alteration in target gene expression. Some examples are Rhll/LasI/PqsA, AhlI, CarI, TraI, CviI, and LuxM synthase, which produces different chain length of acyl lactones that binds to corresponding receptors *Viz*. RhlR/LasRPqsR, AhlR, CarR, TraR, and CviR, respectively (McClean et al., 1997; von Bodman et al., 2003; Dubern and Diggle, 2008; Ng and Bassler, 2009; Nadal Jimenez et al., 2012). Oligopeptides are another class of regulators produced by Gram-positive bacteria, which bind to receptors on cell surfaces and modulate several kinases (Håvarstein et al., 1995). This will lead to alteration in the signal transduction pathway. Like the lactones, AI2 produced by Gram-positive and Gram-negative bacteria produce AI2 that diffuses freely out of the cells (Chen et al., 2002; Neiditch et al., 2006). From the literature survey, it could be easily hypothesized that diverse compounds may regulate signal transduction by modulating signal synthesis, signal perception, degradation, and modifications (Huang et al., 2003; Dong and Zhang, 2005; Chowdhary et al., 2007; Liu et al., 2008). Several reports indicate the use of antibodies to sequester signal molecules, thus limiting their availability to receptors (Kaufmann et al., 2008).

The 3OC12HSL was sequestering using monoclonal antibodies to suppress pathogenic traits in *P. aeruginosa* (Miyairi et al., 2006), while hydrolysis of signal molecules was observed in *P. aeruginosa* (De Lamo Marin et al., 2007; Kapadnis et al., 2009). Inhibition of signal synthesis by binding with enzyme might be one such strategy to control pathogenesis. Triclosan and J8-C8 were competitive synthase inhibitors; thus, the bacteria lower HSL production (Chung et al., 2013). Similarly, pyrogallol, furanone, and canavanine modulate Lux R family receptors, while an anthranilate derivative inhibits PQS synthesis (Givskov et al., 1996; Hentzer et al., 2002; Keshavan et al., 2005; Lesic et al., 2007). Several reports are concerning the modulation of pathogenesis *in vitro*, such as biofilm and pyocyanin inhibition in *P. aeruginosa* and swarming motility inhibition in *S. marcascens* (Marsden et al., 2010; Brackman et al., 2012). Scientists have used nanoparticle-based approaches to control the pathogen, but they are also not economical (Kapadia et al., 2021). Various researchers have reviewed quorum-sensing mediated pathogenesis in the top 10 plant pathogenic bacteria, most of which belong to Gram-negative bacteria (Sibanda et al., 2018). The present study aimed to isolate quorum-sensing inhibitor metabolites from the microbes to target the soft rot-causing pathogen *L. amnigena* RCE. Quorum quenching does not lead to the death of microbes, rather, it suppresses or inhibits the expression of pathogenic traits (Rehman and Leiknes, 2018). The majority of QSI compounds at higher concentration brings about the death of the microorganisms, and their action is mistakenly considered as antibiotic effects. Thus, most bacterial extracts used for identifying bactericidal compounds have not been tested for their ability to regulate quorum-sensing cascade. In the present experiment, *P. aeruginosa* RKC1 produces diverse metabolites and, at higher concentrations, inhibits the growth of Pathogen *L. amnigena* RCE. Quorum-sensing inhibitory activities were recorded using two monitor strains *C. violaceum* MTCC2656 and *P. aeruginosa* MTCC2297, where organic extracts of *P. aeruginosa* RKC1 inhibit purple pigmentation of *C. violaceum* and pyocyanin production as well as swarming motility of *P. aeruginosa* MTCC2297. The quorum-sensing system guides these traits, and the disappearance of such traits indicates the quorum-quenching effects of the metabolites present in the extracts. The metabolites of the extracts disrupt Lux (CviIR) signaling cascade of *C. violaceum* and las, rhl, and pqs signaling system of *P. aeruginosa* (McLean et al., 2004; Sun et al., 2016). *P. aeruginosa* MTCC2297 moved by their flagella in the radial manner from the point of inoculation; likewise, *L. amnigena* also swarms through their peritrichous flagella. The presence of QSI compounds inhibits swarming, and swimming motility indicates their potentiality as quorum quenching. The inhibition of motility by the extracts results in improper cell adherence and is easily approachable. The production of EPS results in the formation of biofilm, which helps the bacteria to adhere to the surfaces for colonization. The biofilm formation by the *L. amnigena* RCE is inhibited by the extracts in a dose-dependent manner. The GC-MS was done to identify the compounds to understand the metabolites responsible for quorum-quenching effects. The data presented here indicate 2,5-diketopiperazines (DKPs) or Cyclic dipeptides such as Cyclo(L-prolyl-L-valine), Cyclo (Pro-Leu), and Cyclo(D-phenylalanyl-L-prolyl) were predominantly found in the organic extracts. These metabolites were found to be the lowest yet detectable for GC-MS in the clear zone around the wells. One can speculate that metabolites might reach the cells and bind with receptors as well as metabolites detected from the zone which cannot correlate with pure extracts. Apart from these several other cyclic dipeptides viz. Cyclo(L-prolyl-L-valine), Cyclo(prolyl tyrosyl), cyclo-(L-Trp-L-Pro), Cyclo-Ala-Pro-diketopiperazine, Cyclo(Leu-Ala), and N-glycylproline found in the extracts at lower peak area and thus unable to be detected in the zone around the wells. Cyclo(Trp-Ser), belongs to *R. aquimaris*, inhibits QS-mediated violacein production in *C. violaceum* and inhibition of biofilm, and pyocyanin production as well as elastase production in *P. aeruginosa* PA01 (Sun et al., 2016). There could be molecular mimicry which leads to either change in the structure of the receptor or make the receptor inaccessible to the natural ligand (Plecha and Withey, 2015). (S)-4,5-Dihydroxypentane-2,3-dione [(S)-DPD, (1)] inhibits quorum sensing and biofilm formation in *V. harveyi* without affecting their growth (Kadirvel et al., 2014). Likewise, cyclic dipeptides and solonomides belonging to *Photobacterium* spp. inhibits the quorum-sensing system of *S. aureus* (Mansson et al., 2011). DKPs were reported to be inhibitors of swarming motilities in *S. liquifaciens* (Holden et al., 1999). The data obtained out of these experiments were supported by the findings of the previous study (Holden et al., 1999), where they showed that cyclo(Ala-Val), cyclo(Pro-Tyr), and cyclo(Phe-Pro) from *P. aeruginosa* inhibits bioluminescence and swarming behavior. Moreover, they also reported that fungi, *Alternaria alternate* derived Cyclo(Pro-Tyr), have the potential to inhibit the swarming motility of *S. liquefaciens*. The molecular docking studies showed that DKPs competitively bind to the receptor, CviR in *C. violaceum* or LasR receptor systems in *P. aeruginosa* PA01 (Holden et al., 1999; Sun et al., 2016; Wang et al., 2022). The analog binding changes the receptor's confirmation, which cannot bind to the target site. Thus, one can speculate that biofilm inhibition and motility reduction in *L. amnigena* RCE could be the results of regulation at the receptor level. The data corroborated with the previous scientific studies where, cyclo(DAla-L-Val), cyclo(L-Phe-L-Pro), and cyclo(L-Pro-L-Tyr) extracted from *P. aeruginosa* inhibits quorum-sensing activator LuxR of *E. coli* (pSB401) by mimicking the AHLs (Jayatilake et al., 1996). They hypothesized that binding of cyclic DKPs to N-terminal domain of LuxR results in the confirmation change at C terminal DNA binding region (Hanzelka and Greenberg, 1995). In the other experiment, DKPs shown to inhibit swarming behavior of *S. liquefaciens* when DKPs were added together with C4-HSL (Eberl et al., 1996). So far, various scientific literatures supported the finding of present experiment (Ortiz-Castro et al., 2011; Borthwick, 2012; Nishanth Kumar et al., 2013; Chen et al., 2015; Wu et al., 2017). There is need of extensive validation at molecular level to elucidate structural changes and binding site. Each pathogen uses different quorum-sensing system to regulate pathogenesis; thus, it is important to evaluate quorum-sensing cascade in them in order to design strategy to combat infection.

## Conclusion

GC-MS analysis of crude extracts from *P. aeruginosa* RKC1 as well as the zone surrounding the wells showed a higher concentration of diketopiperazine (Cyclic dipeptides) like cyclo (L-prolyl-L-valine), cyclo (Pro-Leu), and Cyclo (D-phenylalanyl-L-prolyl). These metabolites, along with other diketopiperazines, account for 93% of the total peak area, suggesting their role as quorum-sensing inhibitors. The extracts showed a noticeable reduction in biofilm inhibition by *L. amnigena* RCE and maceration inhibition on the carrot, potato, and cucumber. This is probably the first report which uses *P. aeruginosa* extracts to control soft rot causing pathogen *L. amnigena* RCE. Furthermore, this diketopiperazine inhibit QS-mediated pathogenicity via competitive binding with receptors. The results substantiate that *P. aeruginosa* extracts could be a potential source for QSI metabolites and can be exploited further at the industrial level. This study opens a novel strategy to control plant pathogens' virulence without developing antibiotic resistance.

## Data availability statement

The original contributions presented in the study are included in the article/Supplementary material, further inquiries can be directed to the corresponding author/s.

## Author contributions

Conceptualization and supervision: CK. Methodology: RK and SS. Formal analysis: KG. Writing—original draft: CK, PP, and RS. Review and editing: RS, PP, SA, MA, and AG. Funding acquisition: SA. All authors have read and agreed to the published version of the manuscript.

## Funding

This project was supported by Researchers Supporting Project Number (RSP-2023R7) King Saud University, Riyadh, Saudi Arabia. The funders had no role in designing and analysis of the results.

## Conflict of interest

The authors declare that the research was conducted in the absence of any commercial or financial relationships that could be construed as a potential conflict of interest.

## Publisher's note

All claims expressed in this article are solely those of the authors and do not necessarily represent those of their affiliated organizations, or those of the publisher, the editors and the reviewers. Any product that may be evaluated in this article, or claim that may be made by its manufacturer, is not guaranteed or endorsed by the publisher.
