# Supplementary material for: Pseudomonas aeruginosa inhibits quorum-sensing mechanisms of soft rot pathogen Lelliottia amnigena RCE to regulate its virulence factors and biofilm formation
Source: Front Microbiol. 2022 Aug 23;13:977669. doi: 10.3389/fmicb.2022.977669 (PMC9450810; doi:10.3389/fmicb.2022.977669)
Supplement: Supplementary file 1 [file Data_Sheet_1.doc]

**Supplementary File**


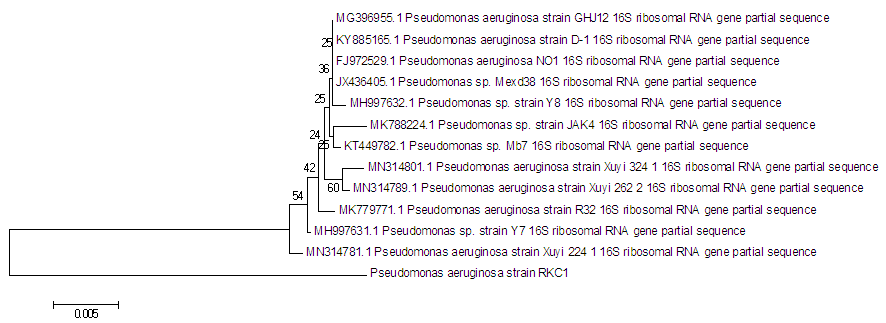


Figure S1: Phylogenetic analysis of *Pseudomonas aeruginosa* RKC1 (RB). Phylogenetic tree showing evolutionary relationship. Nucleotide alignment and Phylogenetic tree was constructed using MEGA7. Numbers at the nodes indicates bootstrap values. Bar represents sequence divergence


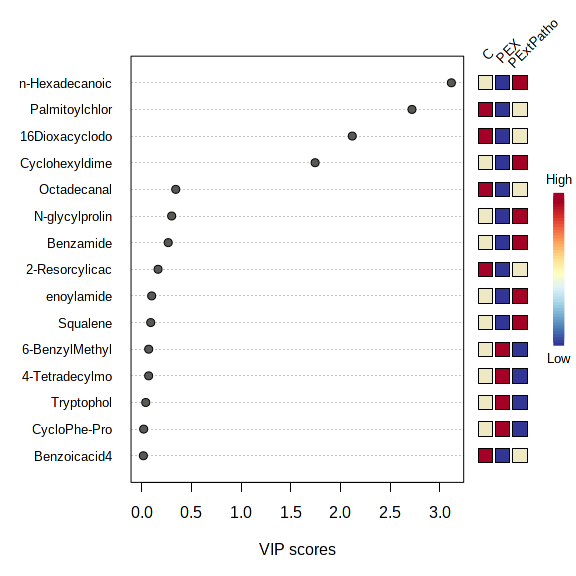


Figure S2: Important features identified by PLS-DA of well diffusion assay. The colored boxes on the right indicate the relative concentrations of the corresponding metabolite in each group under study


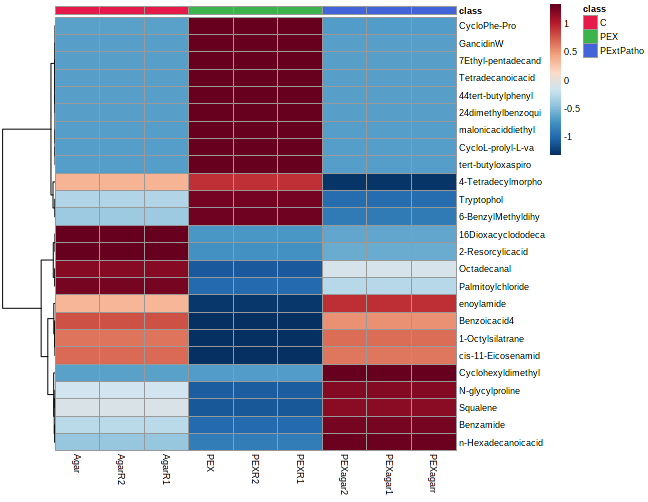


Figure S3: Clustering result shown as heat map of well diffusion assay. (Distance measure using euclidean and clustering algorithm using ward.D)


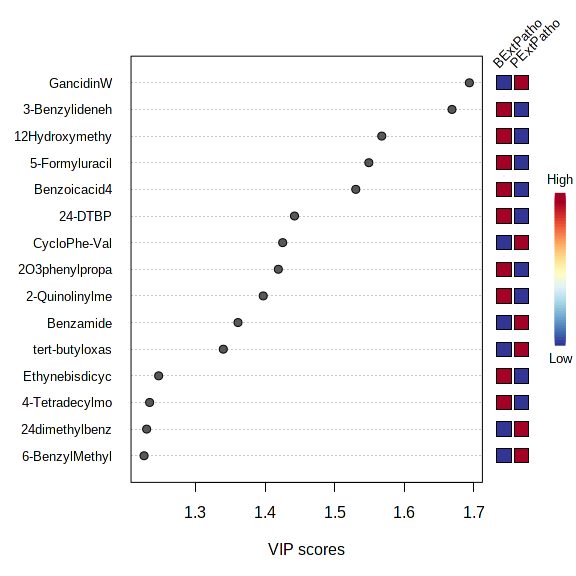


Figure S4: Important features identified by PLS-DA for metabolites regulated in liquid culture assay with bacterial extract. The colored boxes on the right indicate the relative concentrations of the corresponding metabolite in each group under study


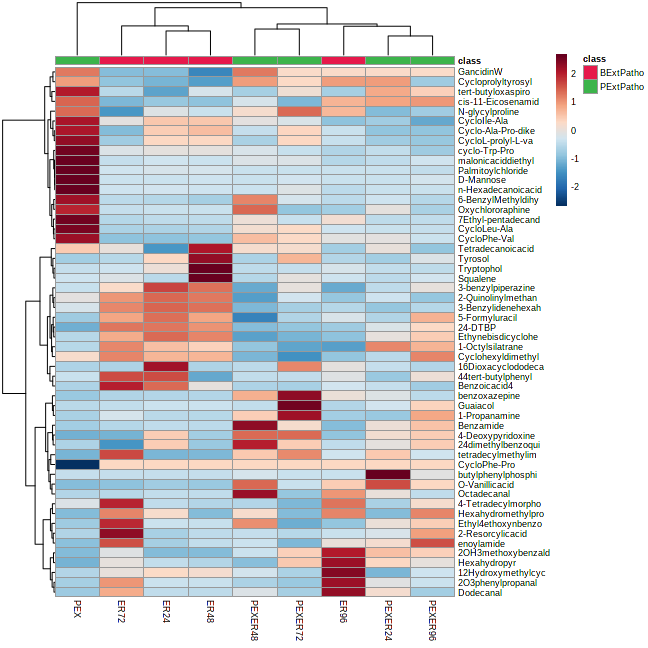


Figure S5: Clustering result shown as heat map of metabolites regulated in liquid culture assay with bacterial extract. (Distance measure using euclidean and clustering algorithm using ward D


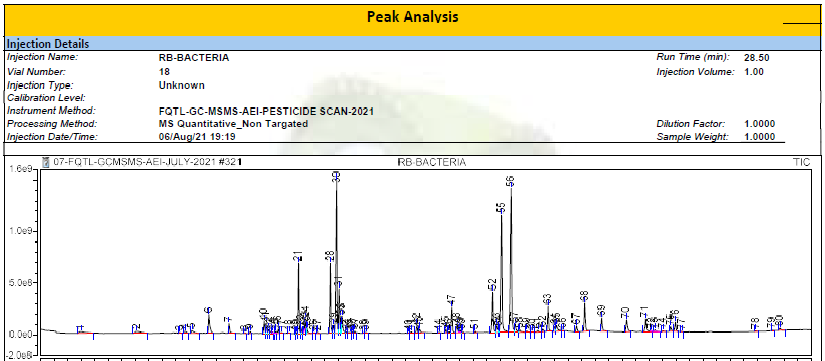


Figure S6: GC-MS spectra of the ethyl acetate extract of RB (*P. aeruginosa* RKC1)
